# A Possible Incomplete Form of Treacher Collins Syndrome: A Case Report

**DOI:** 10.7759/cureus.30203

**Published:** 2022-10-11

**Authors:** Tanmay Tarang, Keta Vagha, Adithya Kiran, Kushagra Singh

**Affiliations:** 1 Paediatrics, Jawaharlal Nehru Medical College, Datta Meghe Institute of Medical Sciences, Deemed University (DU), Wardha, IND; 2 Pediatrics, Jawaharlal Nehru Medical College, Datta Meghe Institute of Medical Sciences, Deemed University (DU), Wardha, IND; 3 Pediatrics and Child Health, Jawaharlal Nehru Medical College, Datta Meghe Institute of Medical Sciences, Deemed University (DU), Wardha, IND

**Keywords:** treacher collins syndrome, retrognathia, genetic disorder, mandibulofacial dysostosis, mandibular hypoplasia

## Abstract

Treacher Collins syndrome (TCS) is a rare genetic disorder that affects craniofacial development due to malformation of the first and second branchial arches. The *TCOF1* gene is mainly responsible for this condition. Here, we present a case of a 13-year-old adolescent girl with complaints of maligned teeth with conductive deafness. On clinical examination, she had retrognathia, a broad nose, maligned teeth, a high arch palate, and midfacial hypoplasia. On the basis of the clinical findings, a diagnosis of a mild-variant TCS was made as eyes were not involved and supportive treatment was given to the patient. The symptoms of the disease have a varying range of severity. Early diagnosis and supportive treatments, which include multidisciplinary treatment involving pediatrics, otolaryngologists, audiologists, orthodontists, and psychologists, are very important for the management of such cases.

## Introduction

Treacher Collins syndrome (TCS) is a rare genetic disease, with 40% family history and 60% de novo mutations. The incidence rate is about 1 in 50,000 live births worldwide [1]. Abnormal craniofacial development due to congenital malformation of the first and second branchial arches occurs in these patients. The extent of abnormalities varies with the severity of the disease. The classical features of TCS may include bilateral malar hypoplasia and mandibular hypoplasia with a prevalence of 81%-97%, coloboma of the lower lid in 54%-69% of the cases, downward slant palpebral fissure in 89%-100% of the cases, and malformation of the external, middle, and internal ear in 83%-92% of the cases [2]. Some of the most apparent symptoms are as follows: slanted eyes with notched lower eyelids and sunken cheekbones and jawbones, pointed nasal prominence, and a large mouth and a high-arched palate. A small fraction of the patients have conductive hearing loss and cleft palate (21%-33%). It is mainly transferred as a de novo mutation, but sometimes, it may be familial. Four different genes responsible for its inheritance are *TCOF1* as autosomal dominant, *POLR1C* as autosomal recessive, *POLR1D* as either autosomal dominant or autosomal recessive, and *POLR1B* as autosomal dominant. In 95% of the cases, *TCOF1* is responsible for it. The diagnosis mainly depends on the clinical features, but confirmation can be made by genetic screening. Patients with less severe TCS are asymptomatic and rarely face any problems. They are retrospectively diagnosed after the birth of a child who has more severe TCS and is symptomatic [3].

Here, we report a 13-year-old adolescent girl who was diagnosed with a case of TCS based on clinical examination when she consulted us for malaligned teeth.

## Case presentation

**Figure 1:**
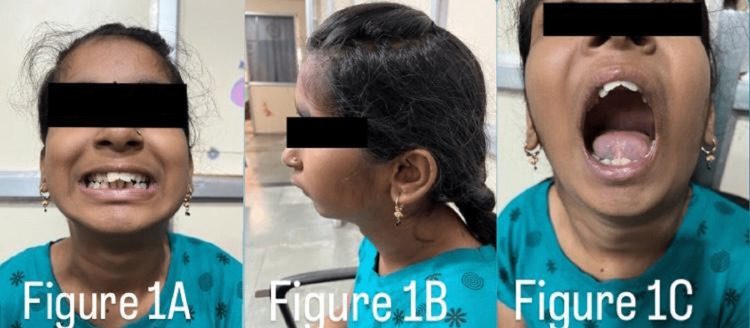
Clinical photographs showing (A) maligned teeth, (B) retrognathia, and (C) a high-vaulted palate.

**Figure 2:**
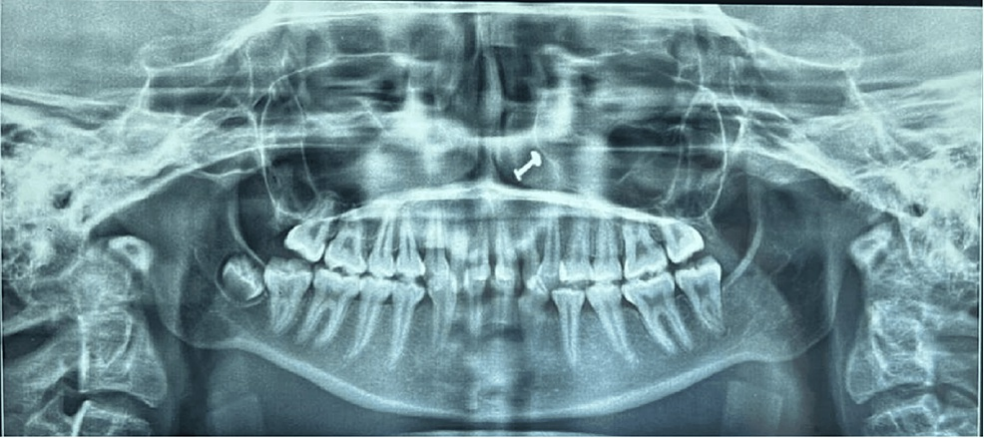
Orthopantomograph of the lower half of the face showing maligned teeth.

**Figure 3:**
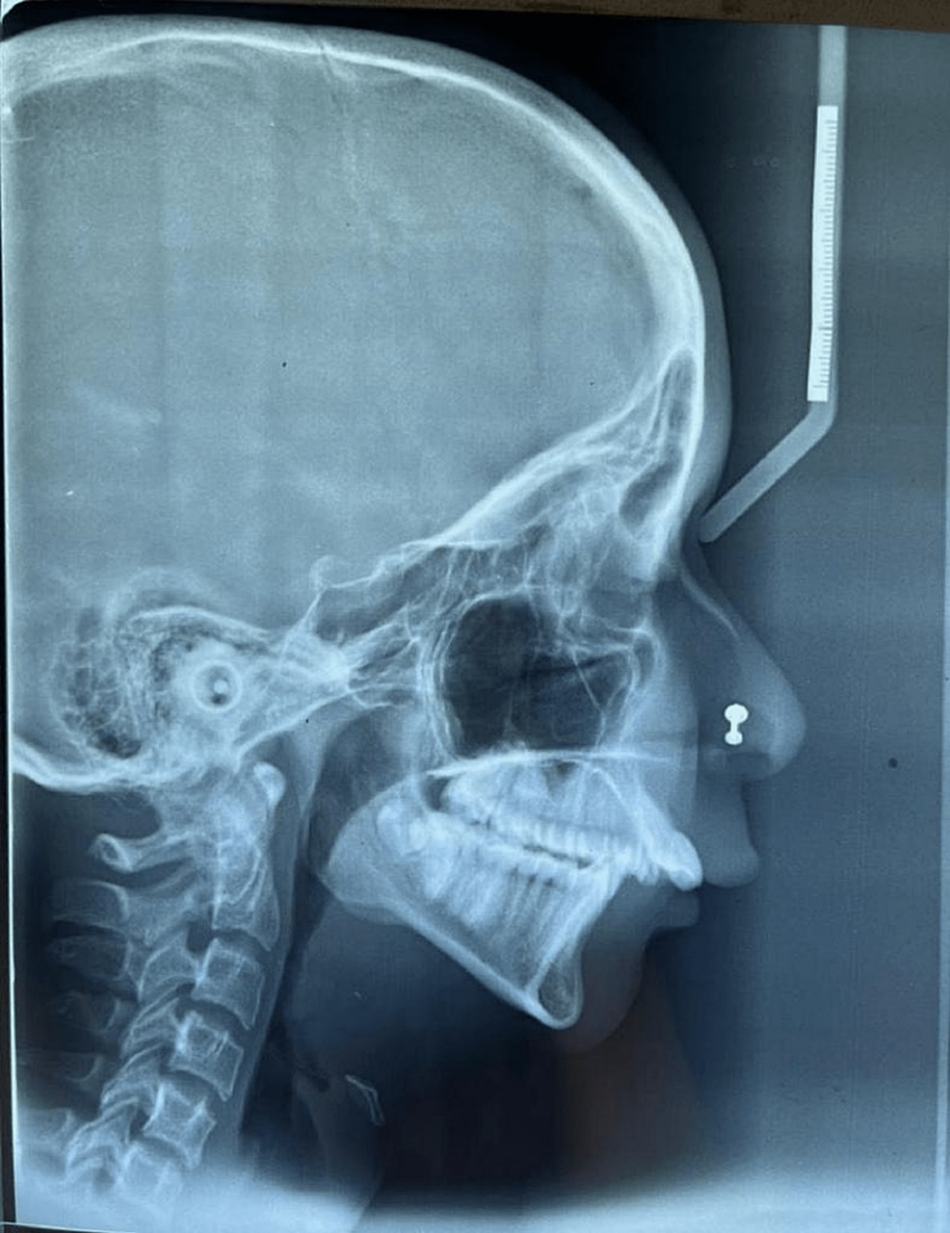
Radiograph of the face in lateral view showing the midfacial hypoplasia in form of underdeveloped nasal bone, maxilla, and mandible.

A 13-year-old adolescent girl who was born out of nonconsanguineous marriage was presented to the hospital with complaints of forward-placed teeth of the upper jaw along with mild hearing loss (Figure 1A). When the patient was examined with the help of craniofacial computed tomography, lateral cephalography, and orthopantomogram, she had midfacial hypoplasia, maligned teeth, a high-arched palate, and a mandible that appeared retrognathic, giving a bird-like appearance (Figure 1B). None of her family members had similar complaints. She had a broad nose and normal eyes. Intraoral examination revealed malocclusion with crowding of maxillary teeth, proclined maxillary incisors, a narrow high-arched palate, absence of third molars, and lateral incisor in the mandible (Figure 1C). A habit of mouth breathing was present. No external ear deformities were observed, but on a hearing test, she had mild conductive hearing loss. She had normal heart functioning with no cardiac abnormalities on physical and clinical examinations. She did not exhibit any intellectual disability. She was operated on for lingual frenulectomy when she was aged two years. The radiographic examination (lateral cephalography) showed a hypoplastic mandible with a prominent antegonial notch with resultant teeth crowding (Figures 2-3), decreased anterior-posterior dimension of the maxilla, and increased height of the lower face. A differential diagnosis of Nager or Miller syndrome, Goldenhar syndrome, and TCS was made. But no limb deformities were present as seen in Nager or Miller syndrome. The deformities were bilateral rather than unilateral, indicating TCS rather than Goldenhar syndrome. Therefore, based on the phenotypic and radiological features, a probable diagnosis of a mild form of TCS was made. Supportive treatment such as hearing aids was given to the patient, and regular follow-ups were suggested for the patient.

## Discussion

TCS is a very uncommon developmental disorder affecting 1 in 50,000 live births. Of the total cases, 60% take place as de novo mutation, whereas 40% as familial [4]. *TCOF1*, which is located on chromosome 5q31.3 32 and codes for the protein serine or alanine-rich nucleolar phosphoprotein necessary for the development of craniofacial, is the critical gene causing this condition [5]. The first and second branchial arches are responsible for forming craniofacial features, and their deformity leads to mandibulofacial dysostosis.

Franceschetti and Klein explained some of the features associated with this syndrome as a downward slanting of the palpebral fissure with either a notch or coloboma of the lower eyelid; hypoplasia of malar, mandibular, and zygomatic bones; abnormal formation of the external, middle, and internal ear; high palate and malposition of the teeth; and some other features such as cleft palate and ears [6]. Hypodontia is also seen in some cases of TCS, primarily affecting the mandibular premolar than the maxillary premolar and incisors. Five clinical forms have been described by a scientist: incomplete, complete, abortive, unilateral, and atypical [6]. The preliminary form, also called incomplete form, may include less severe ear, eye, mandibular, and zygomatic abnormalities, whereas the complete record consists of all known features. The abortive form has only zygomatic hypoplasia and pseudo-coloboma. The unilateral form includes deformities only on one side. The atypical form consists of a combination of other abnormalities that are not usually a part of this syndrome. The clinical feature may vary, and mild cases might go undiagnosed. It is rarely associated with any other disease; however, its association with dermatomyositis was found but failed to explain its pathology [7]. Axelsson et al. named some features for the confirmation of the diagnosis of TCS, which include palpebral fissures with higher temporal corner; abnormality in the lower lid: coloboma of the outer third of the lid, inadequate eyelashes, or both; and hypoplasia of mandible and malar bones [8]. Due to the malformation of the mandibles and maxilla, patients may also complain of oral breathing, which can sometimes worsen sleep apnea. Recently, diagnoses have been made with the help of these obligatory clinical features and genetic screening. There is no exact treatment for TCS. A multidisciplinary approach is often required for the relief of these children, which includes ophthalmological, otolaryngological, dental, surgical, and psychological help. Individuals and their families are recommended for genetic counseling. The absence of a genetic test was a limitation of this study. Some of the differential diagnoses of TCS may include Nager syndrome, acrofacial dysostosis, Goldenhar syndrome, and oculoauriculovertebral spectrum hemifacial microsomia [9]. Nager or Miller syndrome shares the same clinical features of craniofacial deformities, but it also has limb deformities, which are absent in TCS. TCS is different from Goldenhar syndrome as in Goldenhar syndrome, along with facial deformities, cardiac, neural, and respiratory defects are also seen [10].

## Conclusions

TCS is a developmental disorder with characteristic features of craniofacial malformation. The patient typically presents with facial deformities, which range in severities. Early diagnosis of the disease is essential to treat the patient and conduct genetic counseling. Although there is no exact treatment for this condition, symptomatic relief can be provided with a multidisciplinary approach and supportive treatment.
